# The second annual conference of International ovarian cancer consortium and the symposium on tumor microenvironment and therapeutic resistance

**Published:** 2016-01

**Authors:** Ciro Isidoro, Yong Sang Song, Young-Joon Surh, Danny N. Dhanasekaran

**Affiliations:** ^1^ Università del Piemonte Orientale, Department of Health Sciences, Via P. Solaroli 17, Novara, Italy; ^2^ Cancer Research Institute, Department of Obstetrics and Gynecology, Seoul National University College of Medicine, Seoul, Korea; ^3^ Tumor Microenvironment Global Core Research Center, College of Pharmacy, Seoul National University, Seoul, Korea; ^4^ Stephenson Cancer Center, The University of Oklahoma Health Sciences Center, Oklahoma City, OK, USA

**Keywords:** ovarian cancer, tumor microenvironment, therapy resistance, oncogenes, drug discovery

## Abstract

The second Annual Meeting of the International Ovarian Cancer Consortium (IOCC) was held in conjunction with the Symposium on Tumor Microenvironment and Therapeutic Resistance at the Stephenson Cancer Center, University of Oklahoma Health Sciences Center, Oklahoma City, Oklahoma, and USA. A brief welcoming event along with the banquet on Aug 16th was followed by the eight thematic scientific sessions from August 16 to 18, 2015. Forty-three lectures, organized in eight sessions, were discussed in front of an audience of more than hundred attendees. Emphasis was put on oncogene signaling in cancer genesis and progression, new approaches in Precision Medicine and therapy of ovarian cancer, the role of tumor microenvironment in carcinogenesis, and preventive/curative potential of natural products. In this meeting-report, we highlight the findings and the perspectives in cancer biology and therapeutic strategies that emerged during the conference.

## INTRODUCTION

Ovarian cancer remains the leading cause of death from gynaecological malignancies in women, in spite of the progress in drug discovery and improvement in the clinical management of the disease that were made in the last fifty years. Due to non-specific symptoms in the early phases of the disease and the lack of reliable biomarkers, diagnosis of the disease in the early phase has proven to be a formidable challenge. More often than not, the tumor is diagnosed when it is already spread in the peritoneum. Genetic and epigenetic alterations accumulate during progression, that confer heterogeneity to the tumor, giving rise to metastatic and chemoresistant clones. Moreover, the presence of dormant cancer stem cells that show resistance to chemotherapy treatments contributes to the relapse soon after the chemotherapy is terminated. Clearly, to win the battle against ovarian cancer it is mandatory to pool all the expertise available, from cell and molecular biology to bioinformatics, genomics-epigenomics, and clinics.

The International Ovarian Cancer Consortium (IOCC) was founded in 2014 in occasion of its first annual meeting that was held in February 9-11 at the Stephenson Cancer Center (SCC) at the University of Oklahoma Health Sciences Center, Oklahoma City. The Consortium aims to provide a platform for exchanging ideas and facilitating collaborations across the world for a better understanding of the biology and management of ovarian cancer. The scientific program listing the invited speakers and the titles of their lectures delivered at the 1st IOCC meeting is provided at the SCC website. The second meeting of IOCC was held on August 16-18, 2015 in conjunction with the third Symposium on Tumor Microenvironment and Therapeutic Resistance. This choice was quite appropriate, given that the cancer aggressiveness also reflects changes in the composition of the tumor microenvironment, with stromal cells secreting factors that facilitate cancer progression. The Stephenson Cancer Center hosted the event with the grant support from Presbyterian Health Foundation (Oklahoma), and the funding from the NIH COBRE Grant on “Mentoring Translational Cancer Research in Oklahoma (P20 GM103639)”. Other sponsors of the conference are Cancer Research Institute of Seoul National University (Seoul, South Korea), Tumor Microenvironment Research Center of Seoul National University (Seoul, South Korea, the Università del Piemonte Orientale (Italy), Oklahoma Tobacco Settlement Trust, and Oklahoma Medical Research Foundation. Forty-nine invited speakers from nine Countries presented their work in front of more than one hundred delegates. Additional Researchers presented their studies in the Poster session.

More information can be found on the website (http://stephensoncancercenter.org/Research/Research EventsandOther/OklahomaOvarianCancerConference. aspx). Below we summarize the novelties presented in the lectures and the perspectives emerged from the discussion.

### 1 Ovarian Carcinogenesis: Signaling and Stem Cells

As the “SCC-COBRE Distinguished Lecture” speaker, Dr. Premkumar Reddy (Mount Sinai School of Medicine, New York, NY, USA) presented the highlights on his early studies that led to the discovery of the oncogenic mutation in *Ras* and its etiology in tumorigenesis. Dr. Reddy's presentation also focused on his group's recent efforts on targeting Ras-signaling pathways for cancer therapy. Following this, the first session of the conference focused on the origin and progression of ovarian cancer from the cell and molecular biology perspective.

Dr. Jinsong Liu (from the MD Anderson Cancer Center of Houston, Houston, TX, USA) recalled the attention on the polyploid giant cancer cells that can be seen in the context of ovarian cancer. These cells express the typical stem cells markers, such as CD44 and CD133, and may well represent a reservoir of cancer stem cells with a pivotal role in tumorigenesis and in chemoresistance [1]. In the view illustrated by Dr. James Trosko (Michigan State University, East Lansing, MI, USA), the cancer stem cells arise from the exposure to a carcinogen of the natural adult stem cells physiologically present in each organ. The view that ovarian cancer initiates in the peritoneal surface of the ovary epithelium has recently been challenged with alternative hypotheses that also consider a possible colonization of the ovary by cancer cells originating from other organs. Based on the whole genome sequencing of several ovarian carcinomas, Dr. Jeremy Chien (University of Kansas Cancer Center, Kansas City, KS, USA) provided evidence on the origin of ovarian cancer and its rapid progression from an early to a late stage. A phylogenetic analysis of the TP53 mutations and of single nucleotide variations in high grade serous carcinomas showed the presence of ancestral clones in the peritoneal metastases, suggesting that early peritoneal spreading often precedes spreading of ovarian cancer cells [2]. The ovary is continuously (periodically) exposed to hormone stimulations. Dr. Barbara Vanderhyden (University of Ottawa, Ontario, Canada) reported the role of estradiol and progesterone in the initiation and progression of ovarian cancer in a mouse transgenic model. While progesterone was ineffective, the prolonged exposure to estradiol accelerated the initiation and progression of the ovarian carcinogenesis and reduced the survival of tumor-bearing mice through induction of GREB1 expression [3].

Newer insights into oncogenes and tumor suppressors were also presented. Using elegantly developed genetically engineered animal models, Dr. Gloria Su (Columbia University, New York, NY, USA) presented her findings that establish premalignant pancreatic intraepithelial neoplasia (PanINS), pancreatic intraductal papillary mucinous neoplasm (IPMN), mucin-producing precursor neoplasm or mucinous cystic neoplasm are precursors of pancreatic cancer. By comparing the PanIN and IPMN mouse models, her studies provide clues to the intrinsic and extrinsic molecular differences involving p16, p19, p53, TGFβR2, Acvr1b, or Smad4 in addition to mutated K-Ras in pancreatic cancer genesis and progression [4]. Dr. Rakesh Kumar (George Washington University, Washington, DC, USA) presented an overview on the role of MTA1- a member of chromosome remodeling complex in cancer stem cell biology and possibly also in chemo-resistance. Dr. Yung Wong's (Hong Kong University of Science and Technology, Hong Kong, China) presentation emphasized the role of tumor suppressors in regulating tumor progression. Specifically, he described the role of Nm23- family of proteins, specifically Nm23H1, in suppressing H-RasG12V mediated neoplastic transformation and tumorigenesis [5]. Dr. Hye-Kyung Na (Sungshin Women's University, Seoul, South Korea) discussed the role of the inactivation of DNA-methyl transferase 1 in upregulating 15-hydroxyprostaglandin dehydrogenase, a putative tumor suppressor. A novel approach to address the origin and progression of ovarian cancer was presented by Dr. Danny Dhanasekaran (Stephenson Cancer Center, the University of Oklahoma Health Sciences Center, Oklahoma City, OK, USA), whose lecture focused on the involvement of oncogenic long non-coding RNAs. It was found that UCA1, one such l long non-coding RNA, is highly expressed in aggressive ovarian cancer cells and is present in ascitic fluids, and its high expression closely correlates with poor prognosis [6].

### 2 Chemoresistance and Cancer Targeted Therapy

This topic has been addressed in two specific sessions (II and III) and in two special lectures delivered, respectively, by Dr. Premkumar Reddy (see above) and by Dr. Channing Der (Lineberger Comprehensive Cancer Center, Chapel Hill, NC, USA). Dr. Christhardt Koeler (Asklepios Hospital, Hamburg, Germany) and Yong Beom Kim (Bundang Hospital of Seoul National University, South Korea) introduced the theme of clinical management and the strategies to overcome the resistance to paclitaxel and cisplatin in ovarian cancer patients. Dr. Yong Sang Song (Cancer Research Institute, Seoul National University, Seoul, South Korea) examined the role of the ascitic fluid in the progression of ovarian cancer and the onset of chemoresistance. It was found that the acellular fraction of ascitic fluid promoted the expression of genes associated with epithelial-to-mesenchymal transition and favored the invasive behavior of ovarian cancer cells through an IL-6-STAT3 signaling pathway [7]. Other targeted pathways were considered as well. Dr. Anirban Maitra (MD Anderson Cancer Center of Houston, TX, USA), using a whole genome analysis approach, discovered newly emerging therapeutic targets in pancreatic cancer that include the members of COMPASS-like complex and SWI/SNF-family members [8]. Dr. Min Li (University of Oklahoma Health Sciences Center, Oklahoma City, OK, USA) reported his findings on targeting zinc-transporter ZIP4 to overcome therapy resistance in pancreatic cancer. The role of the PI3K- mTOR pathway in ovarian cancer chemoresistance and the combined therapy of the PI3K/mTOR inhibitor BEZ235 together with cisplatin were illustrated by Dr. Yong Li (University of New South Wales, Sidney, Australia). The strategy to overcome drug resistance in breast cancer was presented by Dr. Gary Johnson (Lineberger Comprehensive Cancer Center, Chapel Hill, NC, USA) [9]. The use of monoclonal antibodies against MUC1 (Dr. Li Wang, Zhengzhou University, China) or vascular endothelial cell growth factor (Dr. Debrata Mukhopadhyay, Mayo Clinic Comprehensive Cancer Center, Jacksonville, FL, USA) in the therapy of ovarian cancer also was discussed. Dr. Lali Medina-Kauwe (Cedars-Sinai Medical Center, Los Angeles, CA, USA) presented the development and the therapeutic use of HerPBK10, a recombinant fusion protein based nanoparticle that can penetrate and deliver chemotherapeutic agents to many different human epidermal growth factor receptor (HER)3-expressing tumors. Dr. Ajay Rana (University of Illinois, Chicago, IL, USA) showed that the resistance to Trastuzumab and Lapatinib in HER2-positive breast cancers was associated with reduced expression of the pro-apoptotic Mixed Lineage Kinase MLK-3 [10]. The oncogenic pathway most frequently mutated in human cancer remains, however, the one mediated by Ras, and this explains the efforts so far expended in designing therapies that aim to block the activity of the three Ras family members. The novel promising anti- Ras strategies have been illustrated in the plenary lecture delivered by Dr. Premkumar Reddy and in the distinguished lecture delivered by Dr. Channing Der [11].

### 3 Role of Tumor Microenvironment in Cancer Progression

Cancer cells are embedded in a stroma made up of a glyco-protein matrix containing a variety of host cells that include fibroblasts and immune cells. The secretions of these cells along with the blood-derived molecules constitute a microenvironment that influences the metabolism and the behavior of the cancer cells. The epithelial-stroma communication is bi-directional, as epithelial cancer cells release soluble factors (growth factors, proteases) that modify the stroma composition and *vice versa*. Thus, the cells of the two compartments are subjected to a dynamic reciprocal reprogramming of their metabolism. Understanding the mechanisms that govern such cross-talk may pave the way to new therapeutic approaches. Dr. Maria Diaz-Meco (Sanford Burnham Prebys Medical Discovery Institute, La Jolla, CA, USA) found that the loss of p62, a protein involved in the regulation of the autophagy-lysosomal degradation pathway, in cancer associated fibroblasts favors tumorigenesis because of the altered aminoacid metabolism in the stromal compartment that eventually results in increased production of IL-6 [12]. Interestingly, Dr. Rajagopal Ramesh (Stephenson Cancer Center, the University of Oklahoma Health Sciences Center, Oklahoma City, OK, USA) reported a higher expression of p62 in cisplatin resistant ovarian and lung cancer cells as compared to their respective sensitive counterparts [13]. Consistent with these findings, Dr. Johji Inazawa (Bioresource research Center, Tokyo Medical & Dental University, Japan) found that the high expression of p62 in ovarian cancer cells is associated with poor prognosis [14].

The dynamic (over-time) interactions between all the stromal components (fibroblasts, endothelial and lymphatic cells) and epithelial breast cancer cells have been modeled *in vitro* in 3-D cultures by Bonnie Sloane (School of Medicine of Wayne State University, Detroit, MI, USA), who showed imaging of proteolysis that occurs in tumor microenvironment during cancer cell invasion [15]. Clearly, the tumor microenvironment plays a pivotal role in the reprogramming of mesenchymal stem cells and in maintenance of cancer stem cell features (Dr. Michael Andreff of MD Anderson Cancer Center, Houston, TX, USA). Stromal factors promote angiogenesis, which in turn supports tumor growth and metastasis. Dr. Sukyung Woo (University of Oklahoma Health Sciences Center, Oklahoma City, OK, USA) reported that the resistance to VEGF-targeted agents in ovarian cancer is associated with the activation of the CXCR2-mediated and Apelin pathways. Dr. Asim Abdel-Mageed (Tulane University Health Sciences Center, New Orleans, LA, USA) presented his novel findings that prostate cancer cell derived exosomes that harbor oncogenic small GTPases and several oncomiRNAs are sufficient to induce the neoplastic transformation of pancreatic cancer patient derived adipose stem cells [16].

The inflammatory response and the generation of reactive oxygen species (ROS) in the microenvironment are important players in the development and progression of cancer. Role of redox signaling involving both cytosolic NAD(P)H oxidase and mitochondrial ROS-generating systems in conferring an aggressive invasive phenotype to prostate cancer was discussed by Hari Koul (Louisiana State University Health Sciences Center. Shreveport, LA, USA). David Wink (from National Cancer Institute, NIH, Bethesda, MD, USA) found that the nitric oxide produced in the stroma activates pro-oncogenic signaling pathway in (ER negative breast) cancer cells, while inducing the synthesis of immunosuppressive TGFβ and IL-10 in leukocytes. Consistently, the expression of iNOS (inducible NO synthase) is a strong predictor of poor outcome in patients bearing ER-negative breast cancers [17].

### 4 Bioinformatic and Xenograft Technology for Personalized Treatments

Cancer develops as a mixture of clones that have accumulated multiple genetic and epigenetic changes in individuals who present different genetic background and different history of carcinogen exposure. A big challenge in cancer therapy is to understand the differences and the similarities among cancer cells developing in the same organ of different individuals. Genomic analyses of the transcripts and microRNAs may serve to better delineate the altered pathways that characterize the cancer phenotype. Dr. Pradeep Chaluvally-Raghavan (MD Anderson Cancer Center, Houston, TX, USA) identified two microRNAs, namely miR569 and miR551b, located in the 3q26 amplicon are highly amplified in high- grade serous ovarian cancers and they target proteins controlling apoptosis such as TP53INP1, STAT3, and KIT. Of note, it was found that the overexpression of these miRNAs correlates with poor prognosis in ovarian cancer patients [18]. However, the discovery of complex networks of transcripts and miRNAs that globally control the multiphase process of carcinogenesis and metastasis requires the aid of supercomputer and of dedicated potent software. With this approach, Dr. Satoru Miyano (Human Genome Center, University of Tokyo, Japan) was able to unravel global changes in networks with 13,508 genes related to the EMT phenotype. Focusing on just E-cadherin, he found that as many as 24 genes could control its expression. In addition, the miRNA/mRNA gene network analysis allowed the uncovering of subnetworks of genes controlling drug resistance. Dr. Jonathan Wren (Oklahoma Medical research Foundation, Oklahoma City, OK, USA) focused on bioinformatics-based prediction of useful biomarkers for glioma diagnosis. To this end, he developed an algorithmic approach based upon the integration of a meta-analysis of data from 420,000 human microarray experiments from the literature. The algorithm allowed identifying three potential biomarkers that were validated as highly expressed in high grade gliomas [19]. The diagnostic/prognostic utility of a combination of biomarkers is surely more reliable than a single biomarker. Dr. Tae-Sung Park (Seoul National University, Seoul, South Korea) presented a novel web-based CANcer-specific multimarker Evaluation System (CANES) that allows evaluating the power of candidate markers, using different classifications, across 1,254 molecular expression datasets with 22,624 observations. Another approach to personalized treatment is to test drug sensitivity in mice xenografted with patient- derived tumor that act as surrogates of the patient. This approach was used by Dr. Byoung-Gie Kim (Samsung Medical Center, Seoul, South Korea) to identify potential targets for targeted personalized therapy of ovarian cancer.

### 5 Chemoprevention with Natural Products

Excessive and sustained inflammation causes genomic instability, epigenetic dysregulation and alteration of intracellular signaling pathways. Fighting the generation of ROS in the tumor microenvironment may reduce the burden of genetic and epigenetic changes, thus limiting the progression and expansion of malignant clones. Dr. Duxin Sun (University of Michigan, Ann Arbor, MI, USA) stressed the need for developing new strategies encompassing nanomedicine and natural products to target cancer stem cells. Dr. Shrikant Anant (University of Kansas Cancer Center, Kansas City, KS, USA) reported the ability of gedunin, a heat shock protein (HSP)-90 inhibitor isolated from Indian Neem tree, to inhibit the growth of chemoresistant ovarian cancer cell lines. Dr. Chinthalapalli Rao (Stephenson Cancer Center, University of Oklahoma Health Science Center, Oklahoma City, OK, USA) discussed the anticancer and protective effects of novel and safer nonsteroidal anti-inflammatory drugs. Dr. Doris Benbrook (Stephenson Cancer Center, OUHSC, Oklahoma City, OK, USA) outlined the chemoprevention strategy for ovarian cancer using a newly developed drug, ShetA2, which targets mortalin, a member of HSP-70 family [20]. Dr. Young-Joon Surh (Tumor Microenvironment Global Core Research Center, College of Pharmacy, Seoul National University, Seoul, South Korea) has shown the possibility to dampen the inflammatory reaction in the tumor stroma and to inhibit the activation of signaling pathways associated with the angiogenic and metastastic switches by using resveratrol, a polyphenol found in grapes and berries, among other natural dietary products, as an adjuvant therapy. The chemopreventive and therapeutic properties of resveratrol are largely attributed to the inhibition of the AKT- mTOR anti-apoptotic pathway and to the Nrf2-mediate upregulation of antioxidant enzymes. In addition, Dr. Ciro Isidoro (Università del Piemonte Orientale, Novara, Italy) has shown that resveratrol counteracts the IL-6 inductive invasive phenotype of ovarian cancer cells through the up- regulation of autophagy and ARHI-mediated inactivation of STAT3 signaling pathway [21]. Taken together, these examples highlight the possibility of using natural supplements as complementary treatments to prevent and cure cancer.

## CONCLUSION

The conference promoted the gathering of cancer researchers from more than thirty different institutions from nine different countries, thereby offering the participants with a cooperative network environment for future collaboration. The latest advances in the understanding of the biology of cancer and the possible translational impact on the clinical practice of this knowledge for a better management of cancer patients were successfully discussed among the participants. The conference was adjourned with the resolution that the next annual consortium meeting will be held in Oklahoma City while a satellite meeting will be held in South Korea.
